# Special Dental Journals

**Published:** 1870-04

**Authors:** 


### EDITORIAL DEPARTMENT.

*Special Dental Journals.*—The following is from the "Gynæcological Journal of Boston," edited by Drs. Horatio G. Storer, Winslow Lewis and Geo. H. Bixby:

"In speaking, as we did in our last number, of special medical journals, we purposely deferred till the present moment all comment upon the periodical publications of a department, very important in itself, and which has hardly been appreciated as it should be by the general profession,—we mean Dentistry. Its interest, in its relations to gynæcology, was well pointed by Dr. Hawes in our January number. Dentists cannot always safely decide upon what is best for the jaws of a patient, if ignorant of her pelvic condition, and gynæcologists will often fall far short of a correct diagnosis concerning the causation of neuralgic pains and intestinal disturbance, if they fail to inspect the teeth, as they will of curing the affections coming within their own province, if they neglect to see that the mouth is kept in perfect order.

We take from our table three dental exchanges: the "Ameritan Journal of Dental Science," edited by Dr. Gorgas, and published by Snowden & Cowman, of Baltimore; the "Dental Register," published by Messrs. Taft & Watt, of Cincinnati; and the "Canada Journal of Dental Science," edited by Drs. Chittenden, of Hamilton, and Beers, of Montreal. They are all of them monthlies, are edited with judgement and care, and deserve undoubtedly a generous support. One is struck when examining these periodicals, by the general tone in favor of a conservative surgery. To remove a tooth, as to amputate a leg, is often far easier than to save the member ; in both cases it is the most skilful operator who attempts, where it is possible, to preserve. This would hardly be imagined, however, when visiting a mart of artificial teeth, like that of Codman & Shurtleff, in this city where thousands and tens of thousands of clever counterfeits, almost improvements upon Nature herself, tempt those who would escape dyspepsia or regain the appearance of youth.

To scientific attainments of no mean character, and to mechanical skill, the dentist of the present day must add a knowledge of general medical principles. He must not only be able to use, but to judiciously select, his instruments, often, indeed, himself to fashion them. Of the resources of his profession, one may judge from two books that lie before us: the so-called "Dental Catalogue" of S. S. White, of Philadelphia, elegantly bound and illustrated, and displaying a wonderful fertility of measures for producing comfort by torture, and the "Dental Materia Medica" published by the same firm, also very creditably prepared, and of very evident use,

In this connection, for it is as honorable to his profession as to the individual, we would mention the beautiful volume, entitled "Sanitary Institutions during the Austro-Prussian Conflict" sent us by its author, that eminent dentist, Dr. Thomas W. Evans, of Paris. Whatever the work to which a man devotes himself, if he do it well, he should be duly honored therefor, and though it fall to the lot of but few to become, as Dr. Evans, the titled attendant upon Emperors and an officer of the Legion of Honor, there is no dentist who cannot gain for himself respectful recognition by every medical practitioner, and by the community."

